# Hospital-based headache care during the Covid-19 pandemic in Denmark and Norway

**DOI:** 10.1186/s10194-020-01195-2

**Published:** 2020-10-29

**Authors:** ES Kristoffersen, KW Faiz, EC Sandset, AM Storstein, S Stefansen, BW Winsvold, JM Hansen

**Affiliations:** 1 Department of Neurology, Akershus University Hospital, PO Box 1000, 1478 Lørenskog, Norway; 2 Department of General Practice, University of Oslo, Oslo, Norway; 3 Department of Neurology, Oslo University Hospital, Oslo, Norway; 4 The Norwegian Air Ambulance Foundation, Oslo, Norway; 5 Department of Neurology, Haukeland University Hospital, Bergen, Norway; 6 National Headache Knowledge Center, Rigshospitalet, Copenhagen, Denmark; 7 Department of Research, Innovation and Education, Division of Clinical Neuroscience, Oslo University Hospital, Oslo, Norway; 8 Danish Headache Center, Glostrup – University of Copenhagen, Copenhagen, Denmark

**Keywords:** Migraine, Telemedicine, CGRP, SARS-CoV-2, Health care planning, General population

## Abstract

**Background:**

The Covid-19 pandemic is causing changes in delivery of medical care worldwide. It is not known how the management of headache patients was affected by the lockdown during the pandemic. The aim of the present study was to investigate how the initial phase of the Covid-19 pandemic affected the hospital management of headache in Denmark and Norway.

**Methods:**

All neurological departments in Denmark (*n* = 14) and Norway (*n* = 18) were invited to a questionnaire survey. The study focused on the lockdown and all questions were answered in regard to the period between March 12th and April 15th, 2020.

**Results:**

The responder rate was 91% (29/32). Of the neurological departments 86% changed their headache practice during the lockdown. The most common change was a shift to more telephone consultations (86%). Video consultations were offered by 45%.

The number of new headache referrals decreased. Only 36% administered botulinum toxin A treatment according to usual schemes. Sixty% reported that fewer patients were admitted for in-hospital emergency diagnostics and treatment. Among departments conducting headache research 57% had to halt ongoing projects. Overall, 54% reported that the standard of care was worse for headache patients during the pandemic.

**Conclusion:**

Hospital-based headache care and research was impacted in Denmark and Norway during the initial phase of the Covid-19-pandemic.

## Background

The Covid-19 was declared a pandemic by the World Health Organization (WHO) in March 2020 and led to challenges in health care systems and societies worldwide [1]. In many countries this led to a rapid shift in favor of telemedicine instead of in-person consultations [2–5]. To many patients such a change provided continuous access to care despite infection control measures, but for new-onset headache and complex chronic headache cases, this could result in suboptimal consultations without the possibility of a proper clinical examination or injection treatments [6].

A number of widely used migraine treatments such as nonsteroidal anti-inflammatory drugs (NSAIDs), angiotensin-converting enzyme (ACE) inhibitors, angiotensin II receptor blockers (ARBs) and calcitonin gene-related peptide (CGRP) monoclonal antibodies were all scrutinized for potentially worsening the Covid-19 disease in the initial phase of the pandemic, creating uncertainty among patients and physicians [7, 8]. Impaired access to neurologists may have worsened this situation for many patients. During the pandemic, anecdotal reports emerged describing a considerable drop in the number of headache patients seen in the emergency department and patients treated with injections such as greater occipital nerve block (GON) and botulinum toxin A (BTX) [9, 10]. It was therefore reasonable to fear that a large number of headache patients would miss out on treatment options and would be at increased risk of personal suffering with a corresponding societal socioeconomic impact.

However, to our knowledge studies on this subject have not yet been published. In both Denmark and Norway, nationwide lockdowns due to the pandemic were declared on March 12th 2020.

We hypothesized that the first phase of the pandemic influenced the management of headache. The aim of this “Neurology during a pandemic (NeuroPan) study” was to examine how the lockdown due to the Covid-19 pandemic affected the specialized hospital-based treatment of headache patients in Denmark and Norway.

## Materials and methods

### Design and setting

There are 14 and 18 hospitals with a department of neurology in Denmark and Norway respectively, varying from smaller district hospitals to larger university hospitals. Both countries have a similar population between 5.5 and 5.8 million inhabitants. The structure of the Danish and Norwegian health care system is also similar. The general practitioners (GPs) are reimbursed through a fixed annual fee and additional fees for rendered services from the National Health Insurance. GPs act as gate keepers for referrals to secondary care specialists and hospitals except in emergencies. The hospitals are almost exclusively publicly financed, and both countries have an all-covering national health insurance. Thus, all patients, irrespective of insurance, social or financial status enter the hospital on the same conditions and have the same access to diagnostics, treatment options and further follow-ups. All neurological departments in Norway may prescribe the new CGRP-antibodies for migraine prevention, but only the departments with certified headache clinics (*n* = 6) are allowed to use the treatment in Denmark.

The study was conducted as a questionnaire survey during the lockdown due to the primary stage of the Covid-19 pandemic in Denmark and Norway in 2020. The structured questionnaire about headache treatment was distributed to the Head of headache services at all neurological departments in Denmark and Norway.

### Questionnaire and outcomes

The design of the questionnaire was based on the authors’ clinical experience from the first week of the pandemic lockdown, in addition to their general knowledge and experience in headache medicine and neurology. The questionnaire was distributed in May 2020 and consisted of twenty-four questions of general character concerning their department’s overall handling of headache patients during the initial lockdown period between March 12th and April 15th, 2020.

### Statistical analyses

For descriptive data, proportions, means, and standard deviations (SD), or 95% confidence intervals (CI) are given. Groups were compared using the *t*-test (continuous data) or the *χ*^2^ test (categorical data).

Statistical significance was defined by *p* < 0.05, using a two-sided test. Statistical analyses were performed using IBM SPSS Statistics for Windows, Version 26.00 (SPSS Inc., Chicago, IL, USA).

## Results

The responder rate was 91% (29/32), with 94% (17/18) answering in Norway and 86% (12/14) in Denmark.

Overall, 86% (25/29) of the neurological departments changed their headache practice during the lockdown (Table 1).

**Table 1:** Data on hospital-based headache care during the Covid-19 pandemic in Denmark and Norway (*N* = 29)

	All (N = 29) % (n)		Denmark (*N* = 12) % (n)		Norway (*N* = 17) % (n)	
	Yes	No	Yes	No	Yes	No
The situation and duties at work did change	*86 (25)*	*14 (4)*	*83 (10)*	*17 (2)*	*88 (15)*	*12 (2)*
Our work schedule was changed as a consequence of the pandemic	*41 (12)*	*59 (17)*	*67 (8)*	*33 (4)*	*53 (9)*	*47 (8)*
We reduced the number of beds at the neurology department during the pandemic	*33 (9)*	*67 (18)*	*27 (3)*	*73 (8)*	*38 (6)*	*63 (10)*
Fewer patients with acute headache came to the hospital emergency room for assessment than normal	*60 (15)*	*40 (10)*	*56 (5)*	*44 (4)*	*63 (10)*	*38 (6)*
Have you admitted patients with headache as a primary/debut symptom of Covid-19?	*24 (7)*	*76 (22)*	*33 (4)*	*67 (8)*	*18 (3)*	*82 (14)*
Fewer patients with severe migraine/status migrainosus were admitted during the pandemic than normal	*68 (17)*	*32 (8)*	*56 (5)*	*44 (4)*	*75 (12)*	*25 (4)*
Fewer patients with cluster headache attacks were admitted during the pandemic than normal	*54 (14)*	*46 (12)*	*55 (6)*	*45 (5)*	*53 (8)*	*47 (7)*
We reduced activity at the department’s out-patient clinic during the pandemic	*83 (24)*	*17 (5)*	*83 (10)*	*17 (2)*	*82 (14)*	*18 (3)*
We maintained the usual out-patient clinic for headache patients (with in-person appointments as the norm)	*17 (5)*	*83 (24)*	*8 (1)*	*92 (11)*	*24 (4)*	*77 (13)*
We primarily saw patients for follow-ups and not newly referred patients during the pandemic	*25 (7)*	*75 (21)*	*42 (5)*	*58 (7)*	*13 (2)*	*88 (14)*
We switched to primarily telephone consultations for headache patients	*86 (25)*	*14 (4)*	*83 (10)*	*17 (2)*	*88 (15)*	*12 (2)*
We began offering video consultations for headache patients	*45 (13)*	*55 (16)*	*58 (7)*	*42 (5)*	*35 (6)*	*65 (11)*
Did you refrain from using CGRP antibodies during the pandemic?	*9 (2)*	*91 (21)*	*33 (2)*	*67 (4)*	*0*	*100 (17)*
Did you switch more patients from botulinum toxin to CGRP antibodies than normal during the pandemic?	*21 (5)*	*79 (19)*	*0*	*100 (7)*	*29 (5)*	*71 (12)*
Were you more likely to put patients on CGRP antibodies rather than botulinum toxin as a new treatment for chronic migraine during the pandemic?	*35 (8)*	*65 (15)*	*17 (1)*	*83 (5)*	*41 (7)*	*59 (10)*
If you normally use greater occipital nerve injections, did you continue to do so during the pandemic?	*60 (12)*	*40 (8)*	*71 (5)*	*29 (2)*	*54 (7)*	*46 (6)*
Did your Department continue botulinum toxin treatment for chronic migraine during the pandemic?	*36 (9)*	*64 (16)*	*38 (3)*	*68 (5)*	*35 (6)*	*65 (11)*
Did any of your patients have their treatment aids (O2) revoked or postponed?	*4 (1)*	*96 (25)*	*0*	*100 (9)*	*6 (1)*	*94 (16)*
If you were running research projects, were they halted during this period?	*57 (13)*	*43 (10)*	*50 (4)*	*50 (4)*	*60 (9)*	*40 (6)*
Overall, did headache patients receive the same follow-up as usual during the pandemic?	*38 (11)*	*62 (18)*	*42 (5)*	*58 (7)*	*35 (6)*	*65 (11)*
The overall standard of care for headache patients decreased during the pandemic	*54 (15)*	*46 (13)*	*36 (4)*	*64 (7)*	*65 (11)*	*35 (6)*

Even though only 33% reduced the number of neurological beds during the pandemic, admission rates were decreased for in-hospital investigations and treatments for acute headache (60%), severe migraine/status migrainosus (68%) and cluster headache (54%). There were no significant differences between the countries.

Twenty-four % (7/29) of the hospitals had admitted patients with headache as the debut-symptom of Covid-19.

A total of 83% reduced the out-patient clinic activity during the lockdown. The most common change was a shift to more telemedicine and only 17% maintained the usual in-person out-patient clinic. Telephone consultations were offered by 86%. Video consultations were offered by 45%.

More hospitals in Denmark (42%) than in Norway (13%) focused on follow-up rather than newly referred patients.

**Fig. 1:**
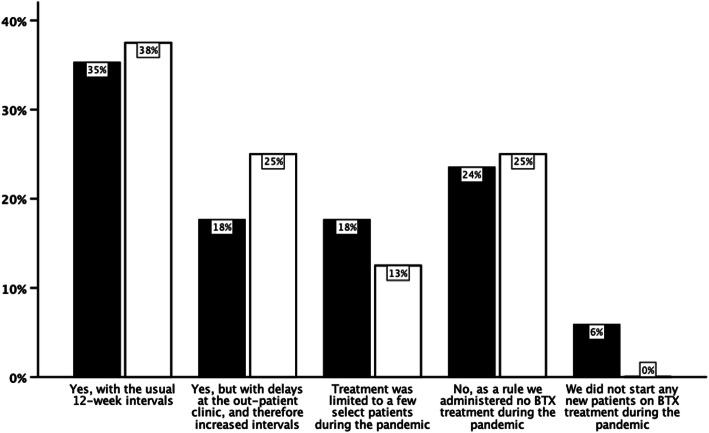
Botulinum toxin A (BTX) treatment in Denmark (white) and Norway (black) during the lockdown. Respondents replied to the question “Did your Department continue botulinum toxin treatment for chronic migraine during the pandemic?”

Most departments routinely used BTX for migraine prevention before the pandemic, except four (14%), all in Denmark. Only 36% of the hospitals offering BTX treatment continued as usual and 28% did not administer BTX at all during the lockdown (Fig. 1). In Denmark no new patients were started on BTX, and only 6% of hospitals in Norway offered new patients BTX. For some patients already on BTX treatment the lockdown led to longer-than usual intervals between treatments (Denmark 25%, Norway 18%) (Fig. 1).

In Norway, 29% of the hospitals reported shifting more patients than normal from BTX to CGRP antibody treatment, whereas no Danish hospitals reported this. In Norway, hospitals were twice as likely to start patients on CGRP antibodies rather than BTX as the new preventive treatment for chronic migraine (41%) compared to Denmark (17%). Of the eligible clinics in Denmark, a third (33%) refrained from using CGRP antibodies during the pandemic, but none of the Norwegian hospitals reported this (Table 1).

Eleven out of 29 hospitals (38%) reported that headache patients received the same follow-up as before the lockdown. Overall, 54% reported that the standard of care was worse for headache patients during the pandemic. A slightly higher proportion of Norwegian hospitals reported worse standard of care compared to Danish hospitals (not statistically significant).

Among departments conducting headache research 57% had to halt ongoing projects.

More than half of the hospitals (55%) acknowledged that the academic community should have collaborated better in creating good solutions for headache patients during the pandemic (Table 2).

**Table 2:** Data on experience with the academic headache community during the pandemic (N = 29)

	All (N = 29)
The academic community should have collaborated better to find good solutions for headache patients during the pandemic.	
Strongly agree	*7 (2)*
Agree	*48 (14)*
Neither agree nor disagree	*38 (11)*
Disagree	*7 (2)*
Strongly disagree	*0 (0)*

## Discussion

The main findings were that 86% of the neurological departments changed their practice during the initial phase of the lockdown and that a worse standard of headache care was reported by more than half of the hospitals. The number of new headache referrals decreased at the out-patient clinics and there was a significant shift to more telephone and video consultations.

During the lockdown, primary care physicians and hospitals expressed concern regarding the impression that fewer people have sought medical help for a number of conditions, including neurological disease. After the pandemic lockdown in the UK there was a 25% fall in emergency room attendances the first [11]. Similar experiences were reported by GPs, the out-of-hours services and primary care emergency wards in Denmark and Norway.

Studies of severe conditions such as acute stroke indicate that the nosocomial threshold was increased during the pandemic [12, 13]. Unfortunately, the present study shows the same pattern for headache patients, as almost 70% of the hospitals reported a decrease in admission rates of severe migraine and 60% reported fewer evaluations for acute headache, normally considered a red flag in headache care [14]. Headache has been reported as a common, but unspecific symptom in both non-hospitalized and hospitalized patients with Covid-19 [15–19]. However, headache is usually not the only symptom of Covid-19, and since most of the hospitalized patients have other and respiratory symptoms, they are usually not referred to neurological departments.

The fact that more than 50% of the hospitals reported fewer admissions with acute cluster headache attacks is surprising as cluster headache attacks are often described as one of the most painful conditions imaginable, with a low threshold for hospitalization [20]. There are no credible reports of fewer patients having severe migraine or cluster attacks during the first weeks of the pandemic. This implies an undertreatment of headache and that fewer patients than normally received proper acute in-hospital treatment.

At more regular times, as few as 2–4% of all headache patients seen by a GP are referred to a neurological department [21]. As most of these patients usually referred to neurologist are highly disabled and have been suffering from headache for many years, it is unlikely that the reduced number of headache referrals are due to changes in the handling by the GPs [22, 23]. However, the decreased number of new headache referrals to the out-patient clinics may be a consequence of reduced access to the GPs or due to an increased patient threshold for seeking medical help during lockdown.

Almost all hospitals reduced the out-patient clinic activity during the lockdown. Only 17% maintained the usual in-person out-patient clinic. Both newly referred patients and already planned follow-ups were postponed in most centres. The most common change was a shift to more telephone consultations (86%). Many headache patients travel far for headache consultations, and a proper treatment plan for non-acute headache can often be achieved without a full neurological examination [24, 25]. Therefore, telemedicine has for a long time been regarded as a potential future appointment option within headache care. A few previous studies have investigated telemedicine in headache care with promising results, but there is a long way from such efficacy studies to a partially forced full-scale implementation in daily practice [26, 27]. Thus, the experiences and long-term effects of virtual consultations for headache patients on a group level should be evaluated after the lockdown.

In addition to the postponed clinical consultations, only 36% of the hospitals continued to administer BTX as before and almost 30% did not administer BTX at all during the lockdown. BTX is one of the few available and effective treatments for a large group of headache sufferers with chronic migraine in Denmark and Norway, and the lack of access to care thus negatively affected the most vulnerable headache patients [9, 28]. Some patients probably endure a delay in the 12-weeks treatment cycles, whereas others are highly vulnerable to changes in the treatment. Furthermore, there is a requirement in Norway that BTX should be effective over three treatment cycles in order to continue receiving the prescription on reimbursement. A delay in treatment may also influence this efficacy evaluation. Chronic headache with its high prevalence has a huge societal impact and economic burden [29, 30]. The utility loss among people with chronic headache is comparable to patients with other chronic diseases, such as chronic obstructive pulmonary diseases, coronary heart diseases and diabetes mellitus [29]. Denied or postponed access to effective treatment options may potentially lead to more sick-leave and consequently high societal costs. Migraine and other headache disorders are associated with low socio-economic status, disability, increased distress and vulnerability [29, 31, 32]. It has been suggested that distressed and vulnerable migraine patients may have even more difficulties in the time of Covid-19 [33, 34]. This may increase barriers towards seeking health care, especially in countries that contrary to Denmark and Norway do not have equal access to health care [35].

Marked difference in the use of CGRP-antibodies emerged between Norway and Denmark. The use of CGRP-antibodies is more restricted and centralized in Denmark, with treatment offered only at the specialized headache centers. This may give advantages in terms of volume per site but also means that patients often have to travel further to get treatment, which in itself may preclude access. During the lockdown access to CGRP-treatment was markedly reduced in Denmark, as 33% of the eligible hospitals did not offer this treatment while treatment with CGRP-antibodies in Norway continued as normal during the lockdown.

In Norway, more hospitals switched patients from BTX to CGRP-antibodies than in Denmark, and Norwegian hospitals were also more likely to start eligible patients on CGRP-antibodies rather than BTX. Collectively, treatment with the new CGRP-antibodies is more accessible in Norway than in Demark, and because it is offered at all neurological departments, less likely to be disrupted by sudden changes as seen during the pandemic and lockdown.

Research projects were halted in most places. This may be justified given the circumstances but pausing intervention trials may yield problems for the patients not receiving the intervention or follow-ups as intended.

In a time with much uncertainty, clinicians may feel an increased need for guidance [36]. Most respondents found the response of the academic community wanting, in the collaboration for better solutions for headache patients during the pandemic.

The fact that hospitals were severely affected by the lockdown underscore that most headache patients can and should be managed in primary care in close collaboration with specialist health care. Strengthening such GP-hospital collaborations would make the headache care less vulnerable in the future. In health care systems where all headache patients are referred or treated by headache specialist at hospitals, a lockdown such as the period in the spring of 2020 will have decisive influence on the possibility of obtaining an effective diagnosis of headache and the continuation of treatment.

As far as we know, this is the first study to report on how the lockdown due to the Covid-19 pandemic affected the management of headache on national levels.

The high responder rate should ensure representativity. The inclusion of two different countries with similar health care systems strengthen the findings. The initial phase of the Covid-19 pandemic was been fairly well controlled in Denmark and Norway. This scenario could of course change, but until now, patients with Covid-19 have not overcrowded the hospitals and by the mid-September 2020 both countries had among the lowest mortality rates reported worldwide [37]. Thus, the handling of headache patients may differ and have more even public health consequences in countries more affected by the pandemic. A potential limitation of the study is the use of the self-reported retrospective questionnaires, however, including all hospitals in two different countries should minimize systematic bias.

## Conclusion

Hospital-based headache care and research was impacted in Denmark and Norway during the initial phase of the Covid-19-pandemic. More research on implementing telemedicine in headache care, institutional and governmental strategies and priorities for headache patients, and headache patients´ overall well-being and prognosis during the long-term lockdown is warranted.

## Data Availability

The authors declare that the data supporting the findings of this study are available within the article.

## Abbreviations

WHO: World Health Organization
NSAIDs: Nonsteroidal anti-inflammatory drugs
ACE: Angiotensin-converting enzyme
ARBs: Inhibitors, angiotensin II receptor blockers
CGRP: Calcitonin gene-related peptide
GON: Greater occipital nerve block
BTX: Botulinum toxin A
GPs: General practitioners
SD: Standard deviations
CI: Confidence interval

## References

[1] Heymann DL, Shindo N (2020). COVID-19: what is next for public health?. Lancet.

[2] Grossman SN, Han SC, Balcer LJ, Kurzweil A, Weinberg H, Galetta SL (2020). Rapid implementation of virtual neurology in response to the COVID-19 pandemic. Neurology..

[3] Hong Z, Li N, Li D, Li J, Li B, Xiong W (2020). Telemedicine during the COVID-19 pandemic: experiences from Western China. J Med Internet Res.

[4] Mann DM, Chen J, Chunara R, Testa PA, Nov O (2020). COVID-19 transforms health care through telemedicine: evidence from the field. J Am Med Inform Assoc.

[5] Roy B, Nowak RJ, Roda R, Khokhar B, Patwa HS, Lloyd T (2020). Teleneurology during the COVID-19 pandemic: a step forward in modernizing medical care. J Neurol Sci.

[6] Szperka CL, Ailani J, Barmherzig R, Klein BC, Minen MT, Halker Singh RB (2020). Migraine Care in the era of COVID-19: clinical pearls and Plea to insurers. Headache..

[7] MaassenVanDenBrink A, de Vries T, Danser AHJ (2020). Headache medication and the COVID-19 pandemic. J Headache Pain..

[8] Arca KN, Smith JH, Chiang C-C, Starling AJ, Robertson CE, Halker Singh RB (2020). COVID-19 and headache medicine: a narrative review of non-steroidal anti-inflammatory drug (NSAID) and corticosteroid use. Headache..

[9] Ali A (2020). Delay in OnabotulinumtoxinA treatment during the COVID-19 pandemic-perspectives from a virus hotspot. Headache..

[10] Silvestro M, Tessitore A, Tedeschi G, Russo A (2020). Migraine in the time of COVID-19. Headache..

[11] Thornton J (2020). Covid-19: a&E visits in England fall by 25% in week after lockdown. BMJ..

[12] Butt JH, Fosbøl EL, Østergaard L, Yafasova A, Andersson C, Schou M et al (2020) The Impact of Coronavirus Disease 2019 (COVID-19) on First-time Acute Stroke and Transient Ischemic Attack Admission Rates and Prognosis in Denmark: A Nationwide Cohort Study. Circulation Epub ahead of print 10.1161/CIRCULATIONAHA.120.050173 PMC7497886 32755320

[13] Kristoffersen ES, Jahr SH, Thommessen B, Rønning OM (2020) Effect of COVID-19 pandemic on stroke admission rates in a Norwegian population. Acta Neurol Scand Epub ahead of print 10.1111/ane.13307 PMC7361547 32620027

[14] Do TP, Remmers A, Schytz HW, Schankin C, Nelson SE, Obermann M (2019). Red and orange flags for secondary headaches in clinical practice: SNNOOP10 list. Neurology..

[15] Kaur N, Gupta I, Singh H, Karia R, Ashraf A, Habib A et al (2020) Epidemiological and clinical characteristics of 6635 COVID-19 patients: a pooled analysis. SN Compr Clin Med:1–5. Epub ahead of print 10.1007/s42399-020-00393-y PMC7343407 32838160

[16] Padda I, Khehra N, Jaferi U, Parmar MS (2020) The neurological complexities and prognosis of COVID-19. SN Compr Clin Med:1–12. Epub ahead of print 10.1007/s42399-020-00527-2 PMC7522181 33015552

[17] Bergquist SH, Partin C, Roberts DL, O'Keefe JB, Tong EJ, Zreloff J et al (2020) Non-hospitalized adults with COVID-19 differ noticeably from hospitalized adults in their demographic, clinical, and social characteristics. SN Compr Clin Med:1–9. Epub ahead of print 10.1007/s42399-020-00453-3 PMC7426161 32838186

[18] Uygun Ö, Ertaş M, Ekizoğlu E, Bolay H, Özge A, Kocasoy Orhan E (2020). Headache characteristics in COVID-19 pandemic-a survey study. J Headache Pain.

[19] Trigo J, García-Azorín D, Planchuelo-Gómez Á, Martínez-Pías E, Talavera B, Hernández-Pérez I (2020). Factors associated with the presence of headache in hospitalized COVID-19 patients and impact on prognosis: a retrospective cohort study. J Headache Pain.

[20] Hoffmann J, May A (2018). Diagnosis, pathophysiology, and management of cluster headache. Lancet Neurol.

[21] Latinovic R, Gulliford M, Ridsdale L (2006). Headache and migraine in primary care: consultation, prescription, and referral rates in a large population. J Neurol Neurosurg Psychiatry.

[22] Ridsdale L, Clark LV, Dowson AJ, Goldstein LH, Jenkins L, McCrone P (2007). How do patients referred to neurologists for headache differ from those managed in primary care?. Br J Gen Pract.

[23] Kristoffersen ES, Grande RB, Aaseth K, Lundqvist C, Russell MB (2012). Management of primary chronic headache in the general population: the Akershus study of chronic headache. J Headache Pain.

[24] Müller KI, Alstadhaug KB, Bekkelund SI (2016). Acceptability, feasibility, and cost of telemedicine for nonacute headaches: a randomized study comparing video and traditional consultations. J Med Internet Res.

[25] Begasse de Dhaem O, Bernstein C (2020) Headache Virtual Visit Toolbox: The Transition From Bedside Manners to Webside Manners. Headache Epub ahead of print 10.1111/head.13885 PMC7323436 32562268

[26] Friedman DI, Rajan B, Seidmann A (2019). A randomized trial of telemedicine for migraine management. Cephalalgia..

[27] Müller KI, Alstadhaug KB, Bekkelund SI (2017). A randomized trial of telemedicine efficacy and safety for nonacute headaches. Neurology..

[28] Dodick DW, Turkel CC, DeGryse RE, Aurora SK, Silberstein SD, Lipton RB (2010). OnabotulinumtoxinA for treatment of chronic migraine: pooled results from the double-blind, randomized, placebo-controlled phases of the PREEMPT clinical program. Headache.

[29] Kristoffersen ES, Stavem K, Lundqvist C, Russell MB (2019). Impact of chronic headache on workdays, unemployment and disutility in the general population. J Epidemiol Community Health.

[30] Jensen R, Stovner LJ (2008). Epidemiology and comorbidity of headache. Lancet Neurol.

[31] Ashina S, Bendtsen L, Buse DC, Lyngberg AC, Lipton RB, Jensen R (2017). Neuroticism, depression and pain perception in migraine and tension-type headache. Acta Neurol Scand.

[32] Buse DC, Manack A, Serrano D, Turkel C, Lipton RB (2010). Sociodemographic and comorbidity profiles of chronic migraine and episodic migraine sufferers. J Neurol Neurosurg Psychiatry.

[33] Ma M, Fang J, Li C, Bao J, Zhang Y, Chen N (2020). The status and high risk factors of severe psychological distress in migraine patients during nCOV-2019 outbreak in Southwest China: a cross-sectional study. J Headache Pain.

[34] Al-Hashel JY, Ismail II (2020). Impact of coronavirus disease 2019 (COVID-19) pandemic on patients with migraine: a web-based survey study. J Headache Pain.

[35] Leonardi M, Lee H, van der Veen S, Maribo T, Cuenot M, Simon L et al (2020) Avoiding the banality of evil in times of COVID-19: thinking differently with a biopsychosocial perspective for future health and social policies development. SN Compr Clin Med:1–3 10.1007/s42399-020-00486-8 PMC7462656 32905109

[36] Wells RE, Strauss LD (2020). The value of headache-specific recommendations during COVID-19. Headache..

[37] Norwegian Institute of Public Health (2020). Daily report and statistics about coronavirus and COVID-19.

