# Correction to: Hospital-based headache care during the Covid-19 pandemic in Denmark and Norway

**DOI:** 10.1186/s10194-020-01199-y

**Published:** 2020-11-16

**Authors:** Espen Saxhaug Kristoffersen, Kashif Waqar Faiz, Else Charlotte Sandset, Anette Margrethe Storstein, Simon Stefansen, Bendik Slagsvold Winsvold, Jakob Møller Hansen

**Affiliations:** 1 Department of Neurology, Akershus University Hospital, PO Box 1000, 1478 Lørenskog, Norway; 2 Department of General Practice, University of Oslo, Oslo, Norway; 3 Department of Neurology, Oslo University Hospital, Oslo, Norway; 4 The Norwegian Air Ambulance Foundation, Oslo, Norway; 5 Department of Neurology, Haukeland University Hospital, Bergen, Norway; 6 National Headache Knowledge Center, Rigshospitalet, Copenhagen, Denmark; 7 Department of Research, Innovation and Education, Division of Clinical Neuroscience, Oslo University Hospital, Oslo, Norway; 8 Danish Headache Center, Glostrup – University of Copenhagen, Copenhagen, Denmark

**Correction to: J Headache Pain 21, 128 (2020)**

**https://doi.org/10.1186/s10194-020-01195-2**

Following publication of the original article [1], we were notified that the author names should have been presented in their full given name and last name, not with initials.

The original article has been corrected.
